# Accidental poisoning with Aconitum: Case report and review of the literature

**DOI:** 10.1002/ccr3.2699

**Published:** 2020-02-05

**Authors:** Giuseppe Bonanno, Mariachiara Ippolito, Alessandra Moscarelli, Giovanni Misseri, Rosaria Caradonna, Giuseppe Accurso, Andrea Cortegiani, Antonino Giarratano

**Affiliations:** ^1^ Department of Surgical, Oncological and Oral Science (Di.Chir.On.S.) Section of Anesthesia, Analgesia, Intensive Care and Emergency Policlinico Paolo Giaccone University of Palermo Palermo Italy

**Keywords:** aconitine intoxication, aconitum, herbal poisoning, ICU

## Abstract

Aconitine intoxication by ingestion of *Aconitum* roots can lead to ventricular tachycardia and cardiac arrest and provides an example of the potential effect of self‐medication. Educational campaigns should be implemented to contain acute intoxications caused by herbal‐derived products.

## Graphical abstract

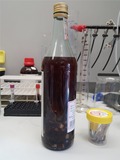

## 1 INTRODUCTION

Herbal medicines are recognized as therapeutic options in alternative and traditional Chinese medicine for several pathological conditions, although improper uses may be harmful. Here, we report a case of a healthy 54‐year‐old man presenting to our emergency department with unexplained ventricular tachycardia (VT), related to *Aconitum* poisoning.

The genus *Aconitum* (Ranunculaceae) consists of hundreds of plant species containing alkaloids, such as aconitine, which have been related to cardiotoxicity, neurotoxicity, and gastrointestinal toxicity.^1^ First described by Fleming in 1845,^2^ *Aconitum* grows in Europe, North America, and Asia and its roots are mainly used in traditional Chinese medicine (TCM) for topical applications to treat musculoskeletal chronic pain.^3^ By now, hundreds of TCM formulations, comprising *Aconitum,* have been described in historical literature and modern clinical reports. Poisoning can be caused by incorrect preparation and application or ingestion of herbal infusions containing *Aconitum* roots derivatives. Main causes of death are refractory ventricular tachyarrhythmias and asystole.^4^ Therapeutic management of *Aconitum* poisoning is supportive and conservative,^5^ as antidotes are not available for this condition.

Here, we describe the clinical management of a case of accidental ingestion of a homemade herbal decoction containing *Aconitum* roots.

## 2 CASE REPORT

A 54‐year‐old Chinese man arrived in our emergency department (ED) for reduced level of consciousness and hypotension. During initial evaluation, the patient underwent cardiac arrest with pulseless ventricular tachycardia (VT) as presenting rhythm, promptly treated with defibrillation. After the prompt return of spontaneous circulation, the patient remained hypotensive (80/60 mm Hg), with a reduced respiratory drive. The anesthesiologist performed intubation and mechanical ventilation and started infusion of noradrenaline and dobutamine; also a lidocaine bolus of 70 mg was given, followed by a continuous infusion protocol. Arterial blood gas showed a metabolic acidosis, with lactate value of 6.2 mmol/L, and no other relevant findings. The patient was subsequently admitted to our intensive care unit.

Patient's clinical conditions appeared to be normal before this apparently unexplained event. Moreover, past medical history was not characterized by comorbidities that might explain hemodynamic impairment. Echocardiogram and coronary angiography showed no abnormal findings. A temporary transvenous pacemaker (PM) was positioned for the onset of episodes of tachyarrhythmias and atrial‐ventricular II type block. Exclusion of acute neurological events was reached by a CT head scan, which did not reveal significant alterations.

**Figure 1:**
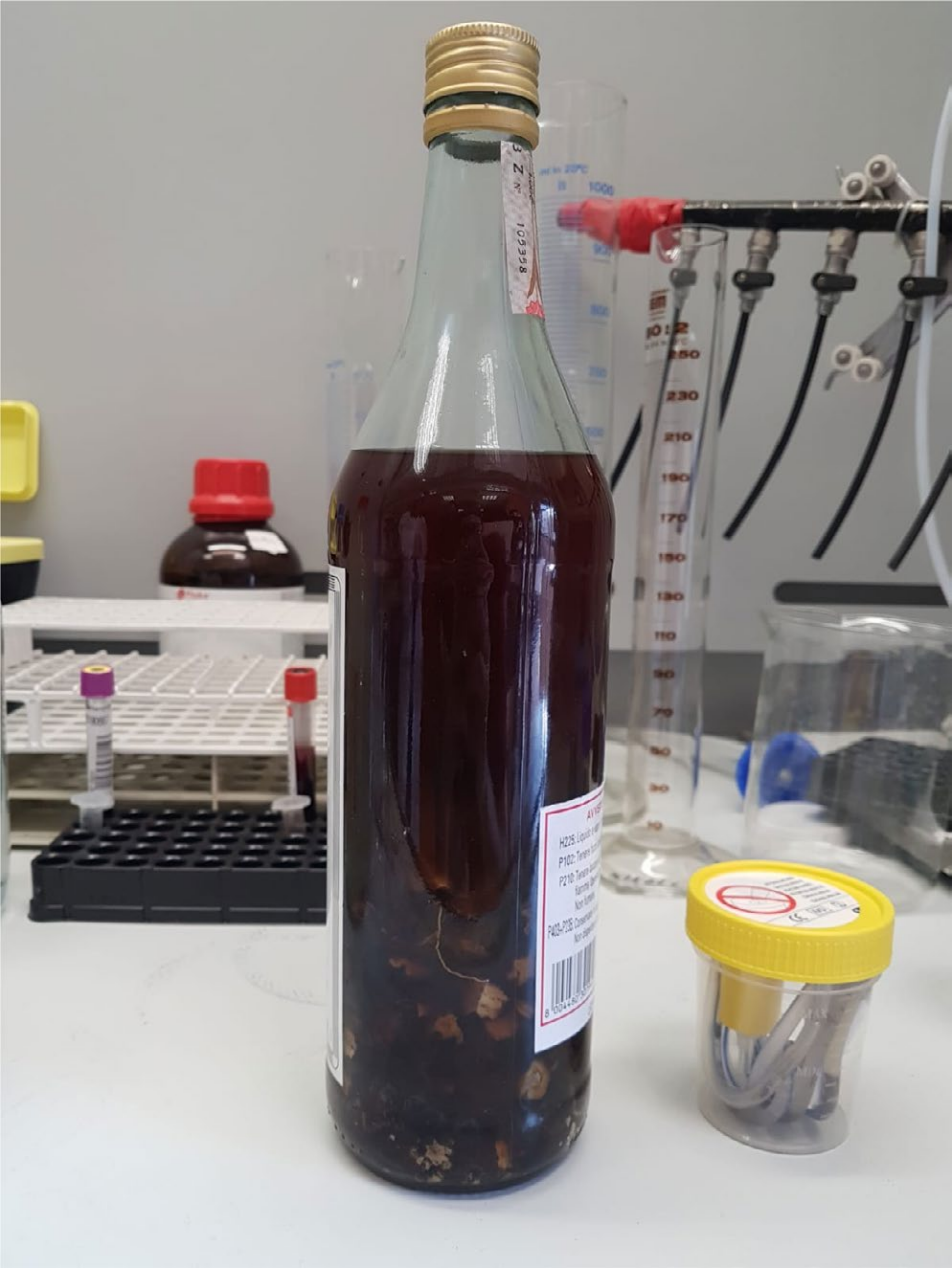
Aconitum bottle provided by the relatives

Deeper investigations on patient daily routine were then conducted, in order to identify possible causes underlying this impaired clinical status. Of note, relatives reported the accidental ingestion of a homemade infusion, which was then identified as an A*conitum* (Figure 1) preparation that the patient had been using as a topical painkiller. As no antidotes are currently available for aconitum poisoning, supportive and conservative management was performed. Supportive therapy determined fast improvement of patient's clinical condition, without the need of charcoal hemoperfusion, although its use was considered by the intensivists involved in the case. On third day of ICU stay, hemodynamic improvements, restoration of sinus rhythm, and efficient respiratory drive allowed PM removal and extubation, with subsequent referral to cardiology department for further examinations, and then discharge without any other sequelae.

## 3 DISCUSSION

Traditional Chinese medicine is an essential part of healthcare system in most Asian countries, and it is based on the use of synergistic properties of multiple herbs and constituents, combined in form of traditional “formulae.” *Aconitum* plants and roots are frequently used for their efficacy in cases of musculoskeletal pain, rheumatic diseases, and abdominal pain.

Unprocessed *Aconitum* can be highly toxic, and its sale is forbidden on the Chinese market. In fact, several types of alkaloids, such as *monoester diterpene* alkaloids, *dieter diterpene* alkaloids (DDAs), and lipoalkaloids, are contained in *Aconitum* plants. Roots may contain high concentrations of these substances, especially when not correctly processed. Although alkaloids are responsible for *Aconitum* analgesic and anti‐inflammatory properties, they cause toxic effects toward myocardium, neurons, and muscle cells.

These toxic properties may be contained by hydrolysis and other chemical manipulations, which decrease their side effects.^4^

Alkaloids exert their actions interacting with Na+ channels, opioid, and serotonin receptors.^6^ Aconitine reaches the α‐subunit of Na+‐channel receptors through the lipophilic part of the membrane and binds the neurotoxin binding site 2, causing cell depolarization and permanent activation of channels themselves. Na+ overload of cardiac cells can induce severe ventricular arrhythmias, like torsade de point or bidirectional ventricular tachycardia.^7^, ^8^, ^9^

Moreover, aconitine modulates noradrenaline and acetylcholine release, favors lipid peroxidation and cell apoptosis,^10^ and has inhibitory effects on neuronal activity in rat hippocampal slices in vitro, and epileptiform activity suppression.^11^

Aconitum poisoning may determine nausea, vomiting, dizziness, palpitation, hypotension, arrhythmias, shock, and coma. Death usually occurs from ventricular arrhythmia within the first 24 hours after intake of *Aconitum* preparations.^4^

Aconitine half‐life has been reported to be about 3 hours,^12^ and intoxication symptoms may persist for 30 hours.^13^

In vitro experiments have demonstrated human skin is also permeable to aconitine and mesaconitine, and poisoning following topical use is described in literature, especially in case of application on damaged epidermis.^14^

Toxicity is possible at therapeutical doses, and safe dosage is dependent on processing. The lethal dose for adults is about 5 mg, but a dose of 2 mg is often sufficient to cause severe cardiac rhythm disturbances.^15^ Severe poisoning has been caused even by ingestion of 0.2 mg pure aconitine, or by decoctions with 6 g of Aconitum roots.^16^ Traditional Western texts recommended 60 mg of root per dose, and consider lethal the dose of 2 mg of pure aconite or 1 g of aconite plant.^17^

In absence of antidotes, usual management is supportive and in case of treatment failure, blood purification^18^ and extracorporeal support may be beneficial.^19^

Ventricular tachyarrhythmias following *Aconitum* poisoning are rarely responsive to lidocaine,^20^, ^21^ unlike in our case. In a recent review, Coulson et al^22^ describe the management of 65 cases of probable aconitine poisoning resulting in ventricular dysrhythmias, reporting that flecainide or amiodarone seems to be more associated with a return to sinus rhythm than lidocaine and/or cardioversion. Moreover, mexiletine, procainamide, or magnesium sulfate may be used to contain tachyarrhythmias caused by this intoxication. The authors also underline how cardiopulmonary by‐pass might be considered as “time‐buying” strategies to allow more rapid excretion of toxic alkaloids while sustaining vital functions.

Chen et al^23^ reported that inadequately prepared herbs, accidental dispensing, and prescription errors are the main causes for aconitine overdose. Of note, herbal medicines have different and often unpredictable effects, due to lack of standardized protocols in their processing phases.

## 4 CONCLUSIONS

Our case demonstrates how multi‐ethnicity and global spread of alternative medicine, such as TCM, may hamper early identification and correct management of clinical cases, especially when alternative treatment self‐medication strategies are followed by patients. Undoubtedly, educational campaigns and ethnic healthcare integration processes should be implemented to contain adverse events related to alternative medicine. Herbal‐derived products, even if recognized as effective in traditional medicine, should be adequately assessed through standardized protocols, in order to contain adverse events related to their self‐medication use.

## CONFLICT OF INTEREST

All the authors declare no conflict of interest.

## AUTHOR CONTRIBUTIONS

GB: conceived the content, drafted the manuscript, and gave final approval of the version to be published. GB, MI, AM, GM, and RC: critically revised the manuscript and gave final approval of the version to be published. GA, AC, and AG: gave important intellectual content, critically revised the manuscript, and gave final approval of the version to be published.
